# Altered Functional and Structural Connectivity Networks in Psychogenic Non-Epileptic Seizures

**DOI:** 10.1371/journal.pone.0063850

**Published:** 2013-05-22

**Authors:** Ju-Rong Ding, Dongmei An, Wei Liao, Jinmei Li, Guo-Rong Wu, Qiang Xu, Zhiliang Long, Qiyong Gong, Dong Zhou, Olaf Sporns, Huafu Chen

**Affiliations:** 1 Key Laboratory for Neuroinformation of Ministry of Education, School of Life Science and Technology, University of Electronic Science and Technology of China, Chengdu, PR China; 2 Department of Psychological and Brain Sciences and Program in Cognitive Science, Indiana University, Bloomington, Indiana, United States of America; 3 Department of Medical Informatics and Medical Image, College of Biomedical Engineering and Medical Imaging, Third Military Medical University, Chongqing, PR China; 4 Department of Neurology, West China Hospital of Sichuan University, Chengdu, PR China; 5 Center for Cognition and Brain Disorders and the Affiliated Hospital, Hangzhou Normal University, Hangzhou, PR China; 6 Faculty of Psychology and Educational Sciences, Department of Data Analysis, Ghent University, Ghent, Belgium; 7 Department of Radiology, West China Hospital of Sichuan University, Chengdu, PR China; Newcastle University, United Kingdom

## Abstract

Psychogenic non-epileptic seizures (PNES) are paroxysmal behaviors that resemble epileptic seizures but lack abnormal electrical activity. Recent studies suggest aberrant functional connectivity involving specific brain regions in PNES. Little is known, however, about alterations of topological organization of whole-brain functional and structural connectivity networks in PNES. We constructed functional connectivity networks from resting-state functional MRI signal correlations and structural connectivity networks from diffusion tensor imaging tractography in 17 PNES patients and 20 healthy controls. Graph theoretical analysis was employed to compute network properties. Moreover, we investigated the relationship between functional and structural connectivity networks. We found that PNES patients exhibited altered small-worldness in both functional and structural networks and shifted towards a more regular (lattice-like) organization, which could serve as a potential imaging biomarker for PNES. In addition, many regional characteristics were altered in structural connectivity network, involving attention, sensorimotor, subcortical and default-mode networks. These regions with altered nodal characteristics likely reflect disease-specific pathophysiology in PNES. Importantly, the coupling strength of functional-structural connectivity was decreased and exhibited high sensitivity and specificity to differentiate PNES patients from healthy controls, suggesting that the decoupling strength of functional-structural connectivity might be an important characteristic reflecting the mechanisms of PNES. This is the first study to explore the altered topological organization in PNES combining functional and structural connectivity networks, providing a new way to understand the pathophysiological mechanisms of PNES.

## Introduction

Psychogenic non-epileptic seizures (PNES) are paroxysmal behaviors, such as involuntary movement, sensation, or experience, which resemble epileptic seizures, but are not accompanied by abnormal electrical activity in the brain [1], [2]. PNES occur often in the general population, with an estimated prevalence of 2-33/100,000 persons per years [3]. The patients with PNES are frequently misdiagnosed and treated for epilepsy, which is detrimental because of the side effects of antiepileptic drugs and the delay in proper treatment [4], [5]. Etiologically, dissociative or conversion disorder is an important manifestation of PNES [6]. Although aetiology facilitated the pathophysiology of PNES, the condition is still mysterious, and only a few lines of evidence from multiple modalities have specifically indicated underlying distributed cortical abnormalities. Specifically, an EEG synchronization study revealed decreased prefrontal and parietal synchronization in PNES, reflecting dysfunction of fronto-parietal networks [7]. More recently, a resting-state functional MRI (fMRI) study implied increased functional connectivity between emotional, executive control and sensorimotor networks in PNES [8]. Together, these studies point to an aberrant functional connectivity in specific brain networks. Little is known, yet, about changes in whole-brain large-scale functional and structural connectivity network in patients with PNES.

Analysis of functional connectivity networks can be conducted via temporal correlation between neural or blood oxygen level-dependent functional MRI signals arising from distinct brain regions [9], [10], thus leading to a network perspective on brain dynamics. Structural connectivity networks, on the other hand, mainly based on white matter tracts quantified by diffusion tractography [11], give insights into microstructural white matter architecture. A network-level assessment of brain function and structure provides a useful and new framework to examine complex network properties of the intact and the diseased brain [12]. Recently, both functional and structural connectivity networks have been found exhibiting small-world architecture [10], [11], [13], allowing global and local parallel information processing [14]. Small-world network reflects two fundamental principles in human brain: functional segregation and integration [15]. Functional and structural connectivity networks are complementary. Structural connectivity network, at least to some extent, is the physical substrate of functional connectivity network [16], [17]. Overall, findings have demonstrated that structural connections are highly predictive of and place constraints on functional interactions across the human brain network over various spatial scales [16], [18]. Conversely, functional connections exert effects on structural connections through mechanisms of plasticity [19]. Evaluating the relationship (coupling) between functional and structural connectivity has got more and more attention, which should greatly enhance our understanding of normal and abnormal mechanism of brain networks [16]. Recent studies have found that the coupling of functional-structural connectivity strengthens with age during development [19], and becomes disrupted in disease states [20], [21]. Accordingly, the coupling of functional-structural connectivity may allow for a more sensitive detection of subtle brain pathophysiological abnormalities than any single modality.

The study of large-scale functional and structural brain networks employing tools and measures of graph theory has proven to be particularly appealing for clinical neuroscience, especially for epilepsy, a disorder of cortical network organization [22]. Commonly, fMRI studies have suggested altered small-world topology and a shift towards more random topology in both partial and generalized epilepsy [21], [23], [24]. In this work, we will examine whether the same topological aberrances of functional connectivity network and structural connectivity network can be seen in PNES. If not, we want to determine what specific network topological organization could be used as a potential biomarker to distinguish PNES from epileptic seizures. Furthermore, as functional connectivity network and structural connectivity network are intimately related and share common small-world topological feature, we predict that the pathological state of PNES may alter this relationship, providing new insights into pathophysiological mechanism underlying PNES.

## Materials and Methods

### Participants

Twenty patients with PNES (7 males, mean age: 19.65±7.56 years) and 20 healthy volunteers (8 males, mean age: 21.85±1.70 years) were recruited from Department of Neurology, West China Hospital, Chengdu, China. PNES patients were given definitive diagnoses by experienced neurologists using clinical descriptions of symptoms and long-term video/EEG monitoring, which is consistent with recent recommendations [1], [25]. The inclusion criteria included: 1) at least one single typical episode was recorded by video EEG, and EEG did not show any epileptiform discharge or ictal slowing; 2) patients have no history of neurological disease; 3) patients have no obvious abnormality in routine structural MRI examinations. The exclusion criteria included: 1) patients with neurological comorbidity (e.g. epilepsy); 2) patients with malingering, or any psychiatric disorders (e.g. mood and anxiety disorders, schizophrenia and psychosis). Only patients with a diagnosis of definite PNES were included in the study. Four of 20 patients were taking antiepileptic drugs before the diagnosis of PNES. All drugs were discontinued at least two weeks prior to MRI examination. Demographic and Clinical Characteristics of PNES can be found in Table S1. To ensure all patients’ illness condition as similar as possible, two patients with very long duration of disease (about 18 years) were excluded in this study. The healthy subjects did not have any neurologic or psychiatric disorders, and take any psychotropic medication within the past six months. This study was approved by local Ethics Committee of West China Hospital. Before experimentation, written informed consent was obtained from each subject and for the minors/children participants, parents signed their written informed consent.

### Data Acquisition

All subjects underwent structural, functional and diffusion tensor imaging scanning on a 3T Siemens Trio system (Erlangen, Germany). During data acquisition, subjects were instructed to relax with their eyes closed, and to keep their heads still. Foam padding and earplugs were used to reduce head motion and scanner noise. Functional images were acquired using a single-shot, gradient-recalled echo planar imaging sequence for a total of 205 volumes (repetition time [TR]/echo time [TE] = 2000/30 ms; flip angle = 90; field of view = 240×240 mm^2^; in-plane matrix = 64×64; voxel size = 3.75×3.75×5 mm^3^, no slice gap; 30 axial slices). The diffusion tensor images covering the whole brain were collected using a single-shot spin-echo planar imaging sequence, including 20 volumes with diffusion gradients applied along 20 non-collinear directions (b = 1000 s/mm^2^) and one volume without diffusion weighting (b = 0 s/mm^2^). Each volume consisted of 50 contiguous axial slices (TR/TE = 6800/93 ms; flip angle = 90°; field of view = 240×240 mm2; in-plane matrix = 128×128; voxel size = 1.88×1.88×3 mm3, no slice gap). Additionally, high-resolution T1-weighted anatomical images were also acquired using a magnetization-prepared rapid gradient-echo sequence (TR/TE = 20/3.69 ms; flip angle = 12; field of view = 250×250 mm^2^; in-plane matrix = 320×320; voxel size = 0.78×0.78×1 mm^3^, no slice gap, 128 sagittal slices) for each subject. Data from one patient was discarder due to uncompleted diffusion tensor images, resulting in a final analysis of 17 PNES patients and 20 healthy controls.

### Construction of Brain Network

#### Anatomical parcellation

Using the automated anatomical labeling (AAL) algorithm [26], we parcellated the whole brain into 90 cortical and subcortical regions of interest (45 for each hemisphere, see Table S2).

#### Construction of brain functional connectivity network

Regional mean time series were corrected by regressing six head motion parameters, averaged signals from ventricles and white matter. The residuals of these regressions were temporally band-pass filtered (0.01–0.08 Hz) [9]. For each subject, a temporal correlation matrix (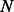
×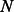, where 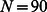 is the number of regions of interest) was obtained by computing the Pearson correlation coefficients between the processed time series of every pair of regions. We constructed weighted functional connectivity networks using absolute functional connectivity strength between connected regions, e.g. 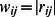, where 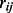 is the correlation coefficient between node 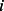 and node 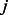.

#### Construction of brain structural connectivity network

For diffusion tensor images, eddy current distortions and head motions were corrected using FMRIB’s Diffusion Toolbox (FSL 4.1; http://www.fmrib.ox.ac.uk/fsl). Then, the diffusion tensor models was estimated by the linear least-squares fitting method at each voxel using the Diffusion Toolkit (TrackVis.org). For each subject, whole-brain fiber tracking was performed in native diffusion space via Fiber Assignment by Continuous Tracking (FACT) algorithm in TrackVis software. To construct a structural connectivity network in each subject, regions of interest were defined in native diffusion space [21]. For edge 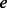, we defined its weight as: 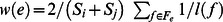. 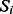 and 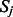 are 2D intersects of the individual’s white matter with region 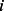 and 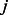; 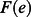 denotes the fibres set connecting regions 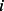 and 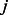; 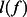 denotes the length of the fiber 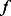. For each subject, connection weights were further scaled by the maximum of this structural connectivity network to normalize individual overall differences in connectivity strength [27]. For more detailed construction of brain network, see Text S1.

### Network Topological Properties

Graph theoretical analysis was employed to compute the topological properties of weighted functional and structural connectivity networks at global and regional (nodal) levels. The global network properties include connectivity strength 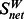, normalized weighted clustering coefficient 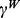, normalized weighted characteristic path length 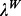 and the small-worldness 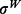. The regional measures include nodal connectivity strength 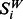, efficiency 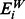 and normalized betweenness centrality 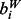. In addition, we also computed these network properties on binarized functional and structural connectivity networks, in which the only difference to weighted networks is that the non-zero weights are set to 1. For network properties, we assigned them with superscripts 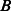 or 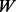 to differentiate binarized and weighted networks, respectively. For definitions and interpretations of these network properties, see Text S1. All network properties were calculated using the Brain Connectivity Toolbox (http://www.brain-connectivtiy-toolbox.net) [28].

### Coupling between Functional and Structural Connectivity Networks

The coupling analysis was constrained by the edges with non-zero structural connectivity, similar to our previous study [21]. The Pearson’s correlation between functional and structural connectivity values was computed to quantify the coupling between functional and structural connectivity networks (Text S1).

### Statistical Analysis

We first computed global topological properties (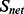, 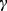, 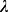, 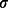) of both weighted and binarized functional and structural connectivity networks for each subject on a range of cost thresholds (0.18≤cost≤0.39). We also calculated the area under the curve (AUC) for global topological properties, which provides a summarized scalar for brain topological properties independent of single threshold selection. Then, a nonparametric permutation test method was performed [27] to detect the significant group differences in global topological properties. A significance threshold of *p*<0.05 (FDR-corrected) was used for testing global topological properties, except AUC of each global topological property for which an uncorrected threshold of *p*<0.01 was used since no multiple comparisons were performed. Of note, before the permutation tests, age and gender for each network properties were regressed out as covariates by multiple linear regression analyses (Text S1).

To detect differences of regional properties, the same permutation framework was performed on the AUC of each regional property (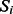, 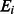 and 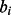). A significance threshold of *p* = 0.01 (uncorrected) was used. To address the problem of multiple comparisons, we set the significance threshold level at *p*<0.01, Bonferroni-corrected for the number of brain regions.

Furthermore, the coupling of functional-structural connectivity was compared by using permutation tests. Testing was performed on the difference of functional-structural connectivity coupling strength.

## Results

### Global Topology of Functional and Structural Connectivity Networks

**Figure 1:**
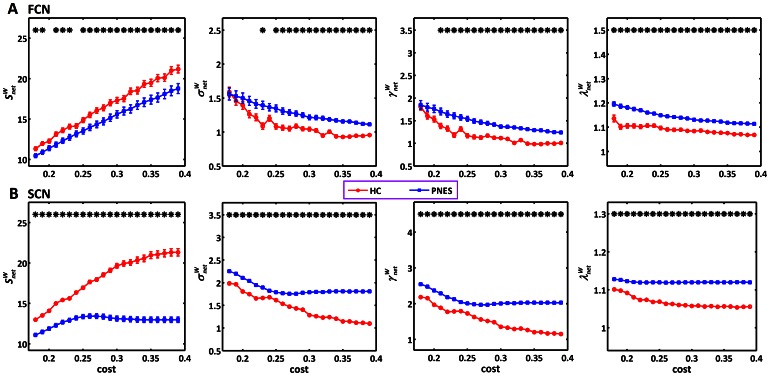
Global network characteristics of weighted brain connectivity network as a function of cost threshold. (A), functional connectivity network. (B), structural connectivity network. From left to right, they are connectivity strength 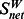, small-worldness 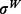, normalized weighted clustering coefficient 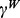 and normalized weighted characteristic path length 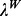. The vertical bar indicates the standard deviation across subjects. The asterisks indicate the statistically significant difference between PNES and healthy controls (*p*<0.05, FDR-corrected). FCN: functional connectivity network; SCN: structural connectivity network; PNES: psychogenic non-epileptic seizures; HC: healthy controls.

**Figure 2:**
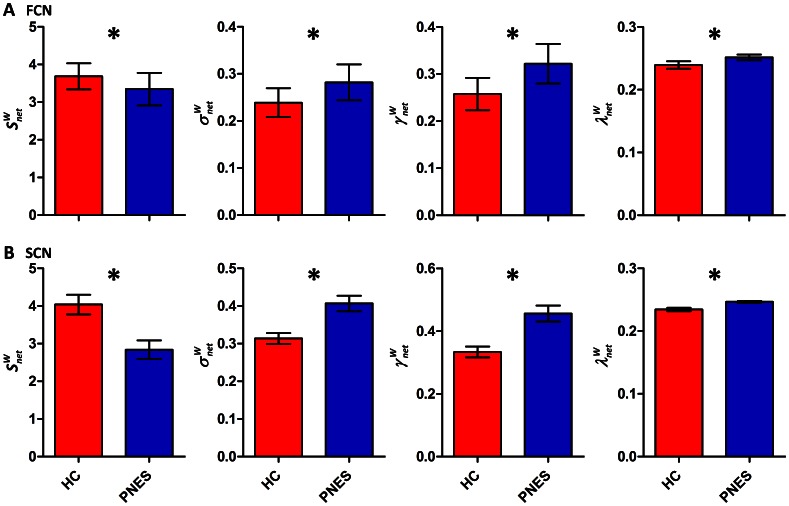
Integrated AUC (area under the curve) of each global network topology in weighted brain connectivity network. (A), functional connectivity network. (B), structural connectivity network. From left to right, they are connectivity strength 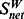, small-worldness 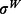, normalized weighted clustering coefficient 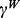 and normalized weighted characteristic path length 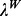. The vertical bar indicates the standard deviation across subjects. The asterisks indicate the statistically significant difference between PNES and healthy controls (*p*<0.01, uncorrected). FCN: functional connectivity network; SCN: structural connectivity network; PNES: psychogenic non-epileptic seizures; HC: healthy controls.

Both PNES patients and healthy controls showed a small-world organization (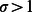) in functional and structural connectivity networks (Fig. 1 and Fig. S1). Additionally, the global topological properties (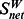, 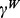, 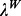, 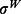) of PNES patients were found to be different from these of healthy controls (*p*<0.05, FDR-corrected). To eliminate the effect of threshold selection, we further compared the integrated AUC of each global network topology and found that the patterns of topological alterations in PNES patients were similar for both functional and structural connectivity networks: decreased network connectivity strength 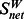, increased normalized weighted clustering coefficient 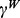, normalized weighted characteristic path length 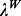 and the small-worldness 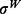 (*p*<0.01, uncorrected) (Fig. 2). For binarized functional and structural connectivity networks, similar results were also found (Fig. S1).

### Regional Alterations in PNES

**Figure 3:**
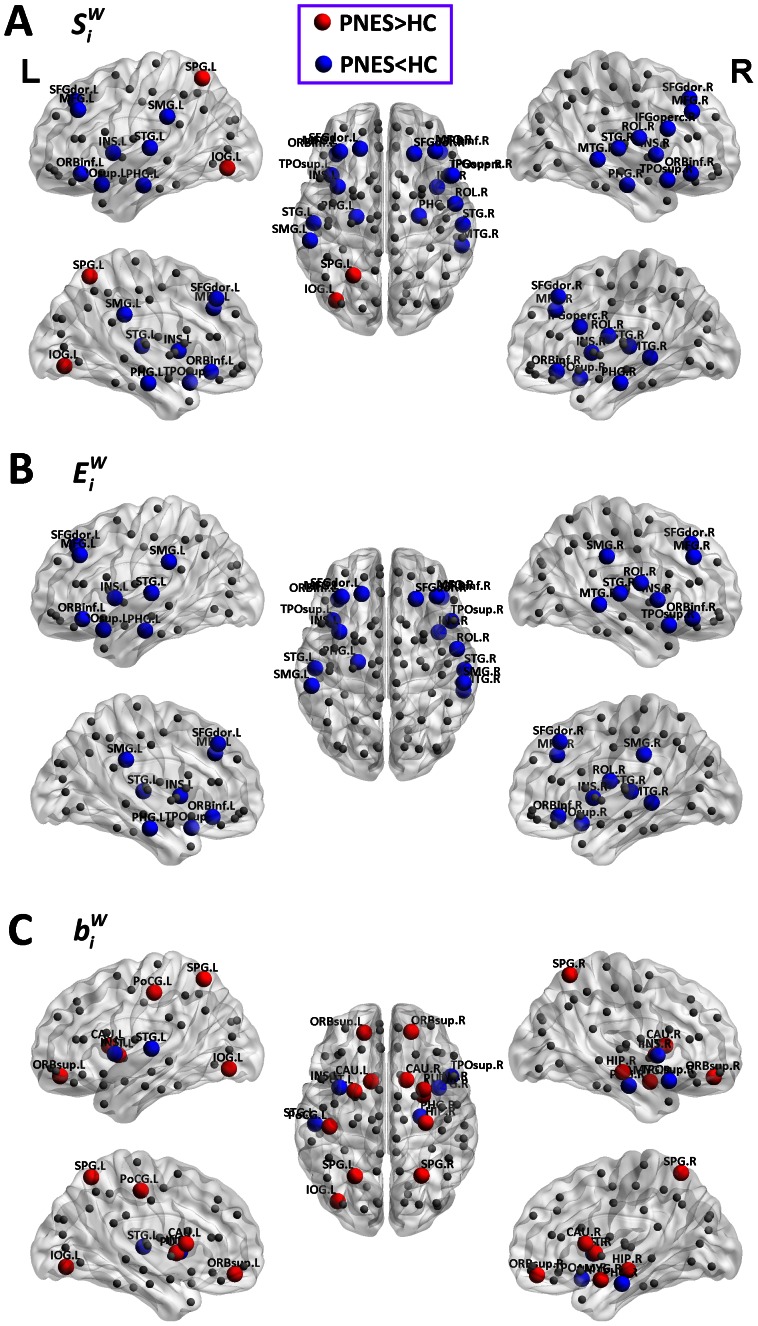
Altered nodal characteristics of weighted structural connectivity network in PNES patient. Results were gained using permutation testing (*p*<0.01, Bonferroni-corrected), and visualized by the BrainNet viewer (NKLCNL, Beijing Normal University). The three dimensional rendering maps show group differences of regional connectivity strength 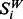 (A), efficiency 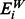 (B), and betweenness centrality 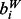 (C). Red/blue spheres indicate regions with increased/decreased nodal characteristic in PNES. Grey spheres indicate regions with no difference. Nodes were positioned according to their centroid stereotaxic coordinates. PNES: psychogenic non-epileptic seizures; HC: healthy controls; L: left; R: right.

Group comparisons on regional connectivity strength 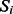, efficiency 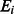, and betweenness centrality 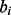 revealed alterations of nodal characteristics in PNES patients, with similar results in weighted and binarized structural connectivity networks (Fig. 3 and Fig. S2). The summary of altered regions can be found in Table 1 and Table S3. Increased 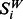 and 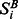 were found in left superior parietal gyrus and inferior occipital gyrus, and increased 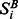 was also found in right superior parietal gyrus and left postcentral gyrus; decreased 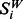 and 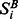 were found in bilateral middle frontal gyrus, orbital part of inferior frontal gyrus, insula, parahippocampal gyrus, superior temporal gyrus, right dorsolateral part of superior frontal gyrus, rolandic operculum, superior temporal pole, middle temporal gyrus, and left supramarginal gyrus, and decresed 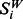 was also found in right opercular part of inferior frontal gyrus, left dorsolateral part of superior frontal gyrus and superior temporal pole (Fig. 3A and Fig. S2A). No regions with increased 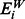 were found, and regions with increased 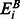 were the same with increased 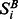. The regions with deceased 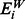 and 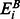 were similar to those in 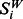, including bilateral dorsolateral part of superior frontal gyrus, middle frontal gyrus, orbital part of inferior frontal gyrus, insula, supramarginal gyrus, superior temporal gyrus, superior temporal pole, right rolandic operculum, middle temporal gyrus, and left parahippocampal gyrus (Fig. 3B and Fig. S2B). Increased 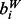 and 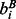 were found in bilateral superior parietal gyrus, putamen, left caudate nucleus, inferior occipital gyrus, and postcentral gyrus, and increased 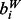 was also found in bilateral orbital part of superior frontal gyrus, right caudate nucleus, hippocampus and amygdala; decreased 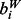 and 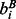 were found in bilateral insula, and decreased 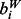 was found in right parahippocampal gyrus, superior temporal pole, and left superior temporal gyrus (Fig. 3C and Fig. S2C).

**Table 1:** Summary of alterations of nodal characteristics in weighted structural connectivity in PNES.

Region	Hemisphere	Strength	Efficiency	Betweenness
SFGdor	L	DEC	DEC	N.S.
	R	DEC	DEC	N.S.
ORBsup	L	N.S.	N.S.	INC
	R	N.S.	N.S.	INC
MFG	L	DEC	DEC	N.S.
	R	DEC	DEC	N.S.
IFGoperc	R	DEC	N.S.	N.S.
ORBinf	L	DEC	DEC	N.S.
	R	DEC	DEC	N.S.
ROL	R	DEC	DEC	N.S.
INS	L	DEC	DEC	DEC
	R	DEC	DEC	DEC
HIP	R	N.S.	N.S.	INC
PHG	L	DEC	DEC	N.S.
	R	DEC	N.S.	DEC
AMYG	R	N.S.	N.S.	INC
IOG	L	INC	N.S.	INC
PoCG	L	N.S.	N.S.	INC
SPG	L	INC	N.S.	INC
	R	N.S.	N.S.	INC
SMG	L	DEC	DEC	N.S.
	R	N.S.	DEC	N.S.
CAU	L	N.S.	N.S.	INC
	R	N.S.	N.S.	INC
PUT	L	N.S.	N.S.	INC
	R	N.S.	N.S.	INC
STG	L	DEC	DEC	DEC
	R	DEC	DEC	N.S.
TPOsup	L	DEC	DEC	N.S.
	R	DEC	DEC	DEC
MTG	R	DEC	DEC	N.S.

L: left; R: right; INC: increase, which means region showing increased nodal characteristic in PNES; DEC: decrease, which means region showing decreased nodal characteristic in PNES; N.S.: no significant, which means region with no significant group difference of nodal characteristic. Results were gained using permutation testing (*p*<0.01, Bonferroni-corrected).

For both weighted and binarized functional connectivity networks, we found no significant differences of nodal characteristics in PNES patients.

### Altered Coupling Strength of Functional-structural Connectivity

**Figure 4:**
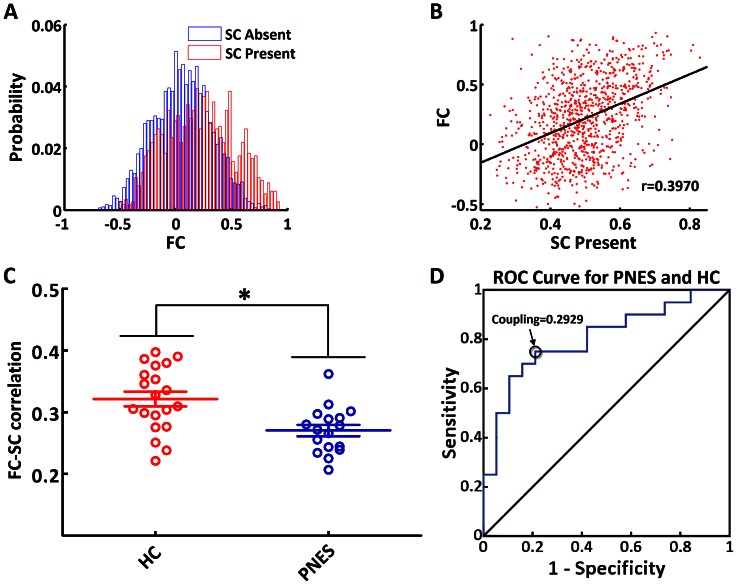
Functional-structural connectivity coupling. (A), the probability densities of functional connectivity values between structurally connected and unconnected region pairs for a selected participant. (B), scatter plot of functional connectivity against non-zero structural connectivity for a selected participant. (C), coupling strength in healthy controls and PNES patients. The asterisks indicate decreased coupling strength of functional-structural connectivity in PNES compared to healthy controls (permutation testing, *p* = 0.0006). (D), a receiver operating characteristic (ROC) curve for PNES and healthy controls. The circle indicates a cutoff point of 0.2929. Using this cutoff value, 13 of 17 PNES patients and 15 of 20 healthy controls were correctly classified, yielding a sensitivity of 75% and a specificity of 77%. The area under the curve for the ROC was 0.78 (95% confidence intervals 0.63 to 0.93). PNES: psychogenic non-epileptic seizures; HC: healthy controls; FC: functional connectivity; SC: structural connectivity.

Whether structural connections are present or not, functional connection values vary over a wide range (Fig. 4A), which is consistent with previous studies [11], [18], [19], [29]. Constrained by existing structural connections, functional connectivity values were positively correlated with structural connectivity values in each participant (Fig. 4B and C), which is similar with previous studies [11], [18], [19]. Compared with healthy controls (0.3213±0.0525), PNES patients (0.2704±0.0377) revealed significant decrease in coupling strength of functional-structural connectivity (t = −3.3305, *p* = 0.0006) (Fig. 4C). Furthermore, receiver operating characteristic (ROC) curves were plotted to explore whether coupling strength values could differentiate PNES patients from healthy controls. As seen in Fig. 4D, the coupling strength correctly classified 13 of 17 PNES patients and 15 of 20 healthy controls, yielding a sensitivity of 75% and specificity of 77%. The area under the curve for the ROC was 0.78 (95% confidence intervals from 0.63 to 0.93), indicating that coupling strength could be used as potential marker for the diagnosis of PNES.

## Discussion

This is the first study to investigate disruptions of network topology and network coupling between functional and structural connectivity networks using graph theoretical analysis in PNES patients. The results revealed that PNES patients had altered small-worldness in both functional and structural connectivity networks, indicating a more regular (lattice-like) organization in large-scale brain networks. Additionally, many regional characteristics were altered in structural connectivity networks, involving attention, sensorimotor, subcortical and default-mode networks. Most importantly, we found that the coupling strength of functional-structural connectivity was significantly decreased and exhibited high sensitivity and specificity to differentiate PNES patients from healthy controls. These results may provide new insights into our understanding of the pathophysiological mechanisms of PNES.

### Altered Global Topology of Functional and Structural Connectivity Networks

The human brain is a large, interacting, complex network with a pronounced small-world topology, which is characterized by high local clustering between neighboring nodes but with short path lengths between any pair of nodes [14]. Small-world topology has been found in both functional and structural networks at either the whole brain or RSNs level [10], [11], [13], which might reflect a fundamental organization principle in the human brain networks [30]. Consistently, our study found that both PNES patients and healthy controls showed small-world topology in functional and structural connectivity networks.

However, compared to healthy controls, PNES patients showed altered topological patterns: increased normalized weighted clustering coefficient 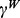 and normalized weighted characteristic path length 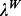, which were similar in functional and structural connectivity networks. Since that the small-world topology reflects an optimal balance between fragmentation and coalescence [14], our results therefore indicate a disturbance of the normal balance in functional and structural connectivity networks in PNES patients. Moreover, the findings of increased local specialization (larger 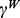) and decreased global integration (larger 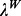) in PNES patients suggest a more regular (lattice-like) organization in large-scale functional and structural connectivity networks. Regular networks have lower ability of global integration and less efficient information propagation compared with small-world networks [31]. Thus, the loss of small-worldness in PNES patients indicates a less optimal topological organization in functional and structural connectivity networks. Currently, there is a high risk to misdiagnose PNES as epilepsy, which not only has serious consequences for the patients because of unnecessary anticonvulsant medication and the delay of treatment, but also has a substantial economic burden for the expensive treatments of intractable epilepsy [4], [5]. It is important to distinguish between PNES and epilepsy. Previous fMRI studies of epilepsy have suggested a shift towards more randomness brain architecture in both partial and generalized epilepsy [21], [23], [24]. In the present study, however, we found a more regular organization of functional and structural connectivity networks in PNES patients, reflecting a different pathogenic mechanism between PNES and epilepsy. This finding might provide a new neuroimaging marker to aid in the diagnosis of PNES.

In addition, we found that the network connectivity strength 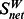 was decreased in both functional and structural connectivity networks in PNES patients. 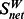 measures the global connectivity strength in the brain networks [11]. The decreased connectivity strength might also suggest ineffective global information processing in PNES patients, since the loss of integration of emotion, executive control and motor function has been found in PNES [8].

### Altered Regional Topology of Functional and Structural Connectivity Networks

In the present study, we investigated the altered nodal characteristics in PNES patients using regional connectivity strength 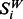, efficiency 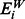, and betweenness centrality 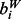. These nodal characteristics can reflect the roles of nodes in information transport and integration across the network [11], [28]. For example, 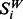 provides information on the total degree of connectivity; 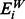 and 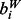 can both quantify the importance of the nodes for the communication within the network. In structural connectivity network, we found that regions with decreased characteristics in PNES patients were mainly involved in attention, sensorimotor and default-mode networks, and increased regional characteristics were mainly in subcortical systems and some other regions.

We observed decreased nodal characteristics in some regions related to attention system (INS, MFG, ORBinf and IFGoperc). The insula is an important multisensory integration area and mediate interpretation of sensory information from the body, which is involved with emotion regulation, visceral sensory perception and self-awareness [32]. The insula and opercular part of frontal gyrus are important regions of attention systems, involving in the coordination and evaluation of task performance [33]. The middle frontal gyrus and orbital part of frontal gyrus were associated with working memory and emotion [34]. Thus, the decreased nodal characteristics of these regions may support that PNES patients are associated with the damage of attentive and emotional function [8].

Decreased nodal characteristics were also found in some regions (SMG, ROL and STG) belonging to sensorimotor network. Sensorimotor network is implicated in self-initiated action as well as in unconscious motor inhibition. PNES are episodes of altered movement and sensation [6]. Recently, Labate et al. [35] investigated structural brain correlates of PNES patients by combining voxel-based morphometry and cortical thickness, and found abnormal cortical atrophy of motor regions. Using resting-state fMRI technique, another study showed that abnormal functional connectivity of PNES was involved in sensorimotor cortex [8]. Combining these findings, our results indicate that sensorimotor network play an important role in the pathogenesis of PNES patients.

In addition, some regions (PHG, MTG, SFGdor and TPOsup) also showed decreased nodal characteristics, which are parts of default-mode networks. Previous studies have found that damage of DMN was associated with a reduced cognitive performance in other brain diseases [36]. Our findings support that PNES are characterized by cognitive dysfunction [7], [35].

Regions with increased 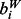 were mainly related to subcortical regions (AMYG, CAU, PUT and HIP). Subcortical regions play an important role in the overall modulation and regulation of emotions and cognition [37]. Damage to subcortical regions will result in a range of impaired behaviors, such as executive dysfunction, memory retrieval deficit and emotional incontinence [37], which are consistent with the clinical manifestations of PNES patients [6]. Considering the decreased nodal characteristics in attention, sensorimotor and default-mode networks, the increased 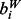 in subcortical regions might indicate a complementary mechanism in PNES.

Taking together, these regions with altered nodal properties suggest dysfunction of emotion, cognitive and motor, supporting the fact that PNES is often accompanied by many diffuse psychological, psychiatric and somatoform symptoms [2]. These regional differences likely reflect disease-specific pathophysiology in PNES.

In the present study, we found no significant differences of nodal characteristics in both weighted and binarized functional connectivity network. This finding is not surprising, since the global network properties in functional connectivity network showed only subtle alterations compared to those of structural connectivity networks. We speculate that the statistically insignificant nodal properties might contribute to these subtle alterations of global measures in functional connectivity network. However, we cannot exclude the possibility that the limited sample size reduced the test power to detect differences of regional characteristics in functional connectivity network in PNES patients.

### Decoupling between Functional and Structural Connectivity Networks

The present study investigated the function-structure relations in large-scale brain networks in PNES patients and healthy controls. Functional network represents temporal coherence of brain regions, and structural network measures anatomical integrity of white matter tracts, the material backbone for communication between brain regions [38]. Structural connections, when present, are highly predictive of, and place constraints on, functional connections. Conversely, functional connections exert effects on structural connections through mechanisms of plasticity [19]. However, structural connections cannot reliably be inferred on the observed functional connections, since strong functional connections with absent structural connections still exist [39]. Perhaps polysynaptic structural connections (in the absence of direct structural connections) enable such functional connections, which results in a complex relationship between functional and structural connectivity. In the present study, we only investigated the function-structure relations on the direct (non-zero) structural connections. Recent studies have found that the function-structure relations can be reconfigured under physiological [18], [19], or pathological states [20], [21].

As we predicted, the coupling of functional-structural connectivity was decreased in PNES patients. This finding suggests loss of coalescence of functional and structural connectomes, reflecting abnormal mechanism of brain networks in PNES patients. Specifically, while taking coupling strength as an index, we could differentiate PNES patients from healthy controls (with a high specificity of 75% and a high sensitivity of 77%). This finding has potential implications for improving the diagnosis and evaluating the treatment of PNES. However, it is difficult to unravel the causality between disrupted functional and structural connectivity networks, which needs to be further examined in future work.

### Study Limitations

Several limitations of the current study should be mentioned. First, the number of patients was relatively small. However, even in this small population, statistically significant alterations in connectivity were found. Second, functional and structural connectivity networks were constructed at a regional level with 90 regions from the AAL atlas. Brain networks with different parcellation schemes or at different spatial scales show distinct network topological organization [40]. Thus, further studies are needed to determine which parcellation strategy or spatial scale is more appropriate to investigate the network mechanism of PNES. Finally, structural connectivity networks were constructed using deterministic tractography, similar to other studies [21]. This may reduce the sensitivity, since the tracking procedure stops when it reaches regions with fibre crossings [41]. In future study, probabilistic tractography techniques [42] should be considered to address this issue.

### Conclusion

In conclusion, we for the first time combined functional and structural connectivity networks to investigate the topological organization of large-scale brain network in patients of PNES. PNES patients lost optimal topological organization in functional and structural connectivity networks reflected by a shift towards more regular brain architecture, which could serve as a potential imaging biomarker for PNES patients. Additionally, structural connectivity networks exhibited altered nodal characteristics in some key regions associated with attention, sensorimotor, subcortical and default-mode systems in PNES. Importantly, we found decreased coupling strength of functional-structural connectivity in PNES, and the coupling showed high sensitivity and specificity to differentiate PNES patients from healthy controls, suggesting that the decoupling strength of functional-structural connectivity might be an important characteristic reflecting the mechanisms of PNES. Our analysis provides a new way to understand the pathophysiological mechanisms of PNES.

## Supporting Information

Figure S1Global network characteristics of binarized functional connectivity network (A) and structural connectivity network (B) as a function of cost threshold. From left to right, they are connectivity strength 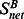, small-worldness 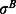, normalized weighted clustering coefficient 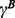 and normalized weighted characteristic path length 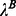. The inset bargraph means integrated AUC (area under the curve) of corresponding network property. The vertical bar indicates the standard deviation across subjects. The asterisks indicate the statistically significant difference between PNES and healthy controls (*p*<0.05, FDR-corrected). The stars indicate the statistically significant difference between PNES and healthy controls (*p*<0.01, uncorrected). FCN: functional connectivity network; SCN: structural connectivity network; PNES: psychogenic non-epileptic seizures; HC: healthy controls.(TIF)

Figure S2Altered nodal characteristics of binarized structural connectivity network in PNES patient. Results were gained using permutation testing (*p*<0.01, Bonferroni-corrected), and visualized by the BrainNet viewer (NKLCNL, Beijing Normal University). The three dimensional rendering maps show group differences of regional connectivity strength 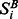 (A), efficiency 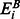 (B), and betweenness centrality 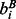 (C). Red/blue spheres indicate regions with increased/decreased nodal characteristic in PNES. Grey spheres indicate regions with no difference. Nodes were positioned according to their centroid stereotaxic coordinates. PNES: psychogenic non-epileptic seizures; HC: healthy controls; L: left; R: right.(TIF)

Table S1 Demographic and Clinical Characteristics of PNES.
(DOCX)

Table S2 Regions of interest (ROI) in the AAL template.
(DOCX)

Table S3 Summary of alterations of nodal characteristics in binarized structural connectivity in PNES.
(DOCX)

Text S1 Supplement methods.
(DOCX)
