# Supplementary figures and images for: Altered Functional and Structural Connectivity Networks in Psychogenic Non-Epileptic Seizures
Source: PLoS One. 2013 May 22;8(5):e63850. doi: 10.1371/journal.pone.0063850 (PMC3661726; doi:10.1371/journal.pone.0063850)

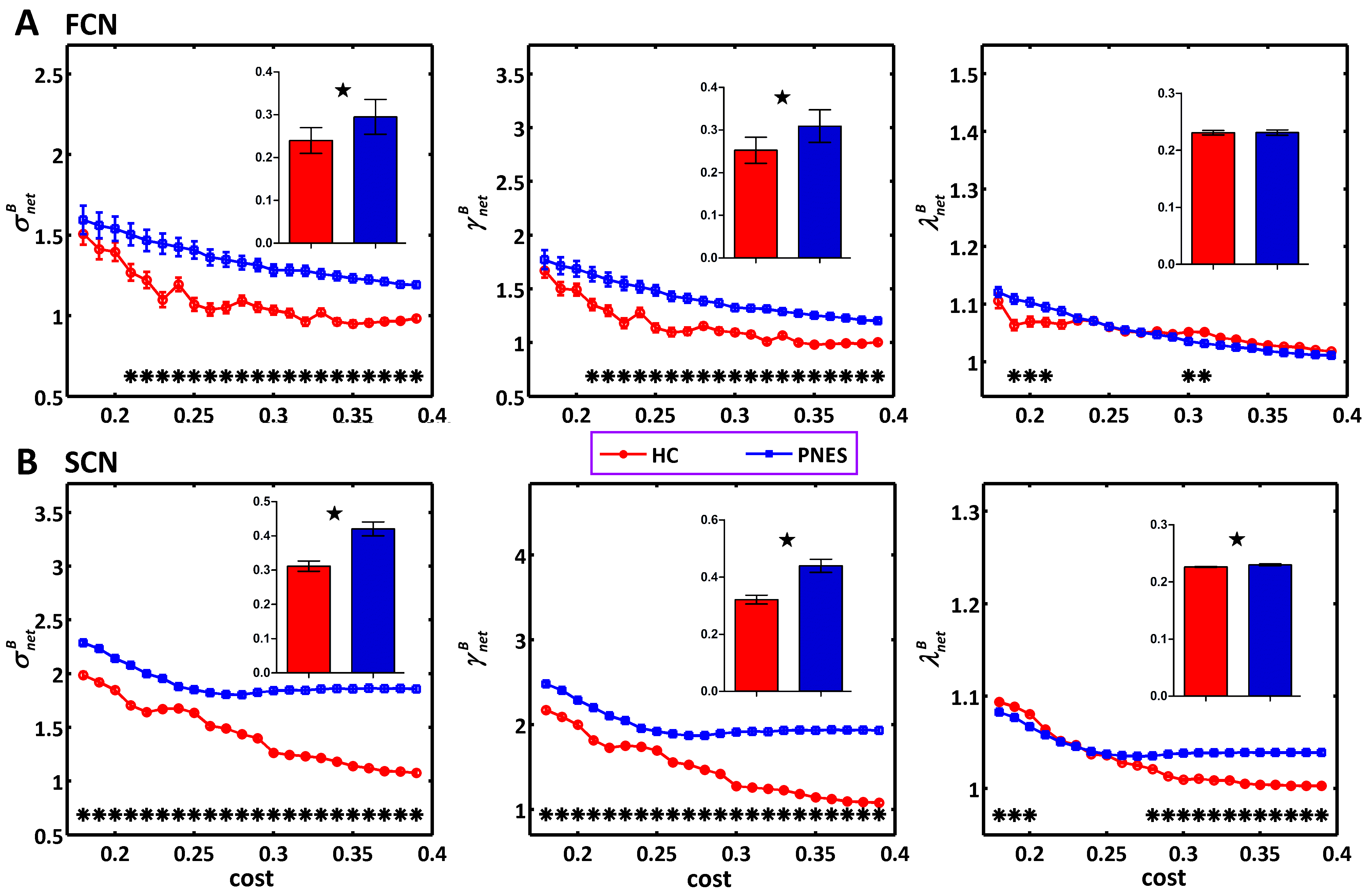

Supplement: Figure S1 — Global network characteristics of binarized functional connectivity network (A) and structural connectivity network (B) as a function of cost threshold. From left to right, they are connectivity strength , small-worldness , normalized weighted clustering coefficient and normalized weighted characteristic path length . The inset bargraph means integrated AUC (area under the curve) of corresponding network property. The vertical bar indicates the standard deviation across subjects. The asterisks indicate the statistically significant difference between PNES and healthy controls (p<0.05, FDR-corrected). The stars indicate the statistically significant difference between PNES and healthy controls (p<0.01, uncorrected). FCN: functional connectivity network; SCN: structural connectivity network; PNES: psychogenic non-epileptic seizures; HC: healthy controls. (TIF) [file pone.0063850.s001.tif]

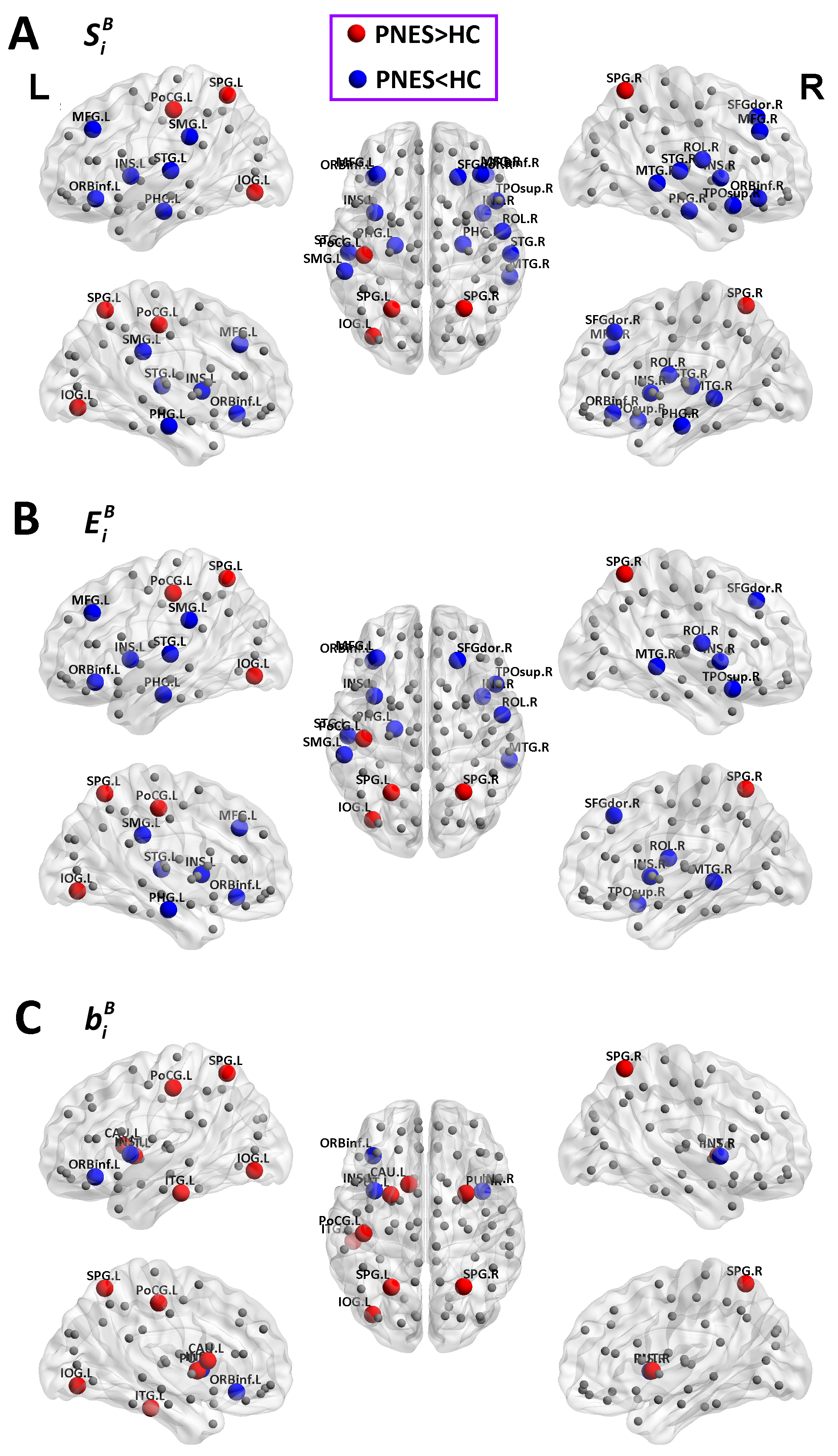

Supplement: Figure S2 — Altered nodal characteristics of binarized structural connectivity network in PNES patient. Results were gained using permutation testing (p<0.01, Bonferroni-corrected), and visualized by the BrainNet viewer (NKLCNL, Beijing Normal University). The three dimensional rendering maps show group differences of regional connectivity strength (A), efficiency (B), and betweenness centrality (C). Red/blue spheres indicate regions with increased/decreased nodal characteristic in PNES. Grey spheres indicate regions with no difference. Nodes were positioned according to their centroid stereotaxic coordinates. PNES: psychogenic non-epileptic seizures; HC: healthy controls; L: left; R: right. (TIF) [file pone.0063850.s002.tif]
