# Supplementary material for: Altered Functional and Structural Connectivity Networks in Psychogenic Non-Epileptic Seizures
Source: PLoS One. 2013 May 22;8(5):e63850. doi: 10.1371/journal.pone.0063850 (PMC3661726; doi:10.1371/journal.pone.0063850)
Supplement: Table S1 — Demographic and Clinical Characteristics of PNES. (DOCX) [file pone.0063850.s003.docx]

**Table S1** Demographic and Clinical Characteristics of PNES

| **Patient** | **Age** | **Gender** | **Duration** | **Type of symptoms** | **Previous Treatment*** |
| --- | --- | --- | --- | --- | --- |
| 1 | 34y | F | 18y | Unresponsiveness/eye closure | None |
| 2 | 17y | F | 2y | Unresponsiveness/eye closure/hyperventilation/bod rigidity | None |
| 3 | 38y | F | 18y | Unresponsiveness/hypermotor EX | None |
| 4 | 23y | F | 8y | Unresponsiveness/eye closure/hyperventilation/bod rigidity | None |
| 5 | 13y | F | 1m | Unresponsiveness/eye closure | None |
| 6 | 20y | M | 2y | Unresponsiveness | VPA |
| 7 | 14y | F | 2m | Unresponsiveness | None |
| 8 | 17y | F | 2m | Unresponsiveness/eye closure/bod rigidity/trembling EX | None |
| 9 | 14y | M | 5m | Unresponsiveness/vocalization | VPA |
| 10 | 16y | F | 2y | Unresponsiveness/hyperventilation/hypermotor EX | None |
| 11 | 17y | F | 4m | Unresponsiveness/hyperventilation | None |
| 12 | 21y | F | 8m | Unresponsiveness/hypermotor EX | None |
| 13 | 21y | M | 1m | Eye closure/hyperventilation/bod rigidity | None |
| 14 | 13y | M | 1y | Unresponsiveness/eye closure/hyperventilation | None |
| 15 | 13y | F | 1y | Unresponsiveness/eye closure/hyperventilation | None |
| 16 | 35y | M | 15d | Unresponsiveness/eye closure/hypermotor EX | None |
| 17 | 16y | F | 3y | Unresponsiveness | CBZ |
| 18 | 20y | M | 2y | Unresponsiveness/hypermotor EX | VPA |
| 19 | 13y | M | 1m | Unresponsiveness | None |
| 20 | 18y | F | 7m | Unresponsiveness/eye closure | None |

*: All the drugs were discontinued at least two weeks before MRI examination.

Abbreviations: F: female; M: male; d: day; m: month; y: year; Hypermotor EX: Hypermotor movements of the extremities; Trembling EX: Trembling of the extremities; VPA: valproate; CBZ: carbamazepine.
