# Supplementary material for: Altered Functional and Structural Connectivity Networks in Psychogenic Non-Epileptic Seizures
Source: PLoS One. 2013 May 22;8(5):e63850. doi: 10.1371/journal.pone.0063850 (PMC3661726; doi:10.1371/journal.pone.0063850)
Supplement: Table S2 — Regions of interest (ROI) in the AAL template. (DOCX) [file pone.0063850.s004.docx]

**Table S2** Regions of interest (ROI) in the AAL template

| **Region name** | **Abbreviation** |
| --- | --- |
| Precentral | PreCG |
| Superior frontal gyrus (dorsolateral) | SFGdor |
| Superior frontal gyrus (orbital part) | ORBsup |
| Middle frontal gyrus | MFG |
| Middle frontal gyrus (orbital part) | ORBmid |
| Inferior frontal gyrus (opercular part) | IFGoperc |
| Inferior frontal gyrus (triangular part) | IFGtriang |
| Inferior frontal gyrus (orbital part) | ORBinf |
| Rolandic operculum | ROL |
| Supplementary motor area | SMA |
| Olfactroy cortex | OLF |
| Superior frontal gyrus (medial) | SFGmed |
| Superior frontal gyrus (medial orbital) | ORBsupmed |
| Rectus gyrus | REC |
| Insula | INS |
| Anterior cingulate gyri | ACG |
| Median cingulate gyri | MCG |
| Posterior cingulate gyrus | PCG |
| Hippocampus | HIP |
| Parahippocampalgyrus | PHG |
| Amygdala | AMYG |
| Calcarine fissure | CAL |
| Cuneus | CUN |
| Lingual gyrus | LING |
| Superior occipital gyrus | SOG |
| Middle occipital gyrus | MOG |
| Inferior occipital gyrus | IOG |
| Fusiform gyrus | FFG |
| Postcentralgyrus | PoCG |
| Superior parietal gyrus | SPG |
| Inferior parietal gyrus | IPG |
| Supramarginalgyrus | SMG |
| Angular gyrus | ANG |
| Precuneus | PCUN |
| Paracentral lobule | PCL |
| Caudate nucleus | CAU |
| Putamen | PUT |
| Pallidum | PAL |
| Thalamus | THA |
| Heschlgyrus | HES |
| Superior temporal gyrus | STG |
| Superior temporal gyrus, temporal pole | TPOsup |
| Middle temporal gyrus | MTG |
| Middle temporal gyrus, temporal pole | TPOmid |
| Inferior temporal gyrus | ITG |

The abbreviations listed are those used in this paper, which differ slightly from the original abbreviations by Tzourio-Mazoyer.
