# Supplementary material for: Altered Functional and Structural Connectivity Networks in Psychogenic Non-Epileptic Seizures
Source: PLoS One. 2013 May 22;8(5):e63850. doi: 10.1371/journal.pone.0063850 (PMC3661726; doi:10.1371/journal.pone.0063850)
Supplement: Table S3 — Summary of alterations of nodal characteristics in binarized structural connectivity in PNES. (DOCX) [file pone.0063850.s005.docx]

**Table S3** Summary of alterations of nodal characteristics in binarized structural connectivity in PNES

| **Region** | **Hemisphere** | **Strength** | **Efficiency** | **Betweenness** |
| --- | --- | --- | --- | --- |
| SFGdor | R | DEC | DEC | N.S. |
| MFG | L | DEC | DEC | N.S. |
|  | R | DEC | N.S. | N.S. |
| ORBinf | L | DEC | DEC | DEC |
|  | R | DEC | N.S. | N.S. |
| ROL | R | DEC | DEC | N.S. |
| INS | L | DEC | DEC | DEC |
|  | R | DEC | DEC | DEC |
| PHG | L | DEC | DEC | N.S. |
|  | R | DEC | N.S. | N.S. |
| IOG | L | INC | INC | INC |
| PoCG | L | INC | INC | INC |
| SPG | L | INC | INC | INC |
|  | R | INC | INC | INC |
| SMG | L | DEC | DEC | N.S. |
| CAU | L | N.S. | N.S. | INC |
| PUT | L | N.S. | N.S. | INC |
|  | R | N.S. | N.S. | INC |
| STG | L | DEC | DEC | N.S. |
|  | R | DEC | N.S. | N.S. |
| TPOsup | R | DEC | DEC | N.S. |
| MTG | R | DEC | DEC | N.S. |
| ITG | L | N.S. | N.S. | INC |

L: left; R: right; INC: increase, which means region showing increased nodal characteristic in PNES; DEC: decrease, which means region showing decreased nodal characteristic in PNES; N.S.: no significant, which means region with no significant group difference of nodal characteristic. Results were gained using permutation testing (*p*<0.01, Bonferroni-corrected).
