# Supplementary material for: Altered Functional and Structural Connectivity Networks in Psychogenic Non-Epileptic Seizures
Source: PLoS One. 2013 May 22;8(5):e63850. doi: 10.1371/journal.pone.0063850 (PMC3661726; doi:10.1371/journal.pone.0063850)
Supplement: Text S1 — Supplement methods. (DOCX) [file pone.0063850.s006.docx]

**Text S1 Supplement Methods**

**Construction of brain network**

**Anatomical parcellation**

To construct large-scale brain functional and structural connectivity networks, we first determine the nodes of brain network. Using the automated anatomical labeling (AAL) algorithm [[1](#_ENREF_1)], we parcellated the whole brain into 90 cortical and subcortical regions of interest (45 for each hemisphere, see Supplemental material Table S2). This parcellation scheme was applied to the following network analyses, which is in line with our previous study [[2](#_ENREF_2),[3](#_ENREF_3)].

**Construction of brain functional connectivity network**

Functional images preprocessing was performed on the Statistical Parametric Mapping software (SPM8, <http://www.fil.ion.ucl.ac.uk/spm>). The first five volumes were not analyzed to allow for signal equilibration effects. The remaining 200 consecutive volumes were corrected for temporal differences and head motion, spatially normalized to the Montreal Neurological Institute (MNI) echo-planar imaging template and resampled to 3-mm cubic voxels. There were no subjects with movement greater than 1.5 mm translation or 1.5° rotation. Recent studies have showed that functional connectivity analysis is sensitive to gross head motion effects [[4](#_ENREF_4),[5](#_ENREF_5)], we further evaluated the group differences in translation and rotation of head motion according to the formula (Liu etal., 2008): $Head\text{ }Motion/Rotatio=1/\left(L-1\right)\sum_{i=2}^{L} \sqrt{\left|x_{i}-x_{i-1}\right|^{2}+\left|y_{i}-y_{i-1}\right|^{2}+\left|z_{i}-z_{i-1}\right|^{2}}$. $L$ is the length of the time series ($L=200$ in this study), $x_{i}$,$y_{i}$ and $z_{i}$ are translations/rotations at the $i$th time point in the $x$,$y$ and $z$ directions, respectively. The results showed that there were no significant differences between patients and healthy controls in head motion and rotation (two-sample two-tailed $t$ test, $t=0.3119$, $p=0.7568$ for translational motion and $t=0.7421$, $p=0.4626$ for rotational motion). For each subject, the mean time series of each region of interest were acquired by averaging the time series across all voxels within that region. Regional mean time series were corrected by a linear regression process to remove several spurious sources of variances, including six head motion parameters, averaged signals from ventricles and white matter. The residuals of these regressions were temporally band-pass filtered (0.01-0.08 Hz) to remove high and low frequency noise sources [[6](#_ENREF_6)], and used to substitute for the raw mean time series of the corresponding regions. For each subject, a temporal correlation matrix ($N$×$N$, where $N=90$ is the number of regions of interest) was obtained by computing the Pearson correlation coefficients between the processed time series of every pair of regions. In order to avoid loss of information, we constructed weighted functional connectivity networks using absolute functional connectivity strength between connected regions, e.g. $w_{ij}=\left|r_{ij}\right|$, where $r_{ij}$ is the correlation coefficient between node $i$ and node $j$.

**Construction of brain structural connectivity network**

For diffusion tensor images, eddy current distortions and head motions were corrected using FMRIB’s Diffusion Toolbox (FSL 4.1; <http://www.fmrib.ox.ac.uk/fsl>). Then, the diffusion tensor models was estimated by the linear least-squares fitting method at each voxel using the Diffusion Toolkit [[TrackVis.org; 7](#_ENREF_7)]. For each subject, whole-brain fiber tracking was performed in native diffusion space via Fiber Assignment by Continuous Tracking (FACT) algorithm in TrackVis software (Wang et al., 2007). Path tracking was terminated if it reached a voxel with fractional anisotropy less than 0.15 or turned an angle greater than 35°, as in our previous study [[2](#_ENREF_2),[8](#_ENREF_8)]. To construct a structural connectivity network in each subject, regions of interest were defined in native diffusion space, as described in previous studies [[2](#_ENREF_2),[9](#_ENREF_9),[10](#_ENREF_10)]. Briefly, T1-weighted anatomical images of each subject were first co-registered to the b0 image in the native diffusion space using a linear transformation. Co-registered T1-weighted images were then normalized to the ICBM-152 MNI T1-template using a nonlinear transformation. The derived transformation parameters were inverted and used to warp the AAL regions from MNI space to the native diffusion space with nearest-neighbor interpolation. In native diffusion space, two regions $i$ and $j$ were connected through an edge $e=\left(i,j\right)$, if there exists at least one fibre $f$ with end-points in both region [[9](#_ENREF_9),[11](#_ENREF_11),[12](#_ENREF_12)]. For each edge $e$, we defined its weight $w\left(e\right)$ as: $w\left(e\right)=2/\left(S_{i}+S_{j}\right)\sum_{f\in F_{e}} 1/l\left(f\right)$, which capturing the connection density between the end-nodes of the edge $e$ [[11-13](#_ENREF_11)]. Here, $S_{i}$ and $S_{j}$ are 2D intersects of the individual’s white matter with AAL region $i$ and region $j$, respectively [[2](#_ENREF_2),[8](#_ENREF_8)]; $F\left(e\right)$ denotes the set of all fibres connecting regions $i$ and $j$; $l\left(f\right)$ denotes the length of the fiber $f$ along its trajectory. As a result, we constructed weighted structural connectivity networks. For each subject, connection weights were further scaled by the maximum of this structural connectivity network to normalize individual overall differences in connectivity strength [[14](#_ENREF_14)].

**Network topological properties**

Graph theoretical analysis was employed to compute the topological properties of functional and structural connectivity networks at global and regional (nodal) levels. Since weighted networks keep the information of connectivity strength giving a better understanding of the network organizations, the present study mainly focused on weighted functional and structural connectivity networks. As a supplementary investigation, we also computed the topological properties of binarized networks, in which the only difference to weighted network is that the non-zero weights are set to 1. For some network properties are differently defined between binarized and weighted networks, we assigned them with superscripts $B$ or $W$ to differentiate the two kinds of networks, respectively. All network properties were calculated using the Brain Connectivity Toolbox (<http://www.brain-connectivtiy-toolbox.net>) [[15](#_ENREF_15)]. All formulas introduced below are based on $G=(N,K)$, a graph of network $G$ with $N$ nodes and $K$ edges.

**Global network properties**

Network connectivity strength $S_{net}$

The connectivity strength is defined as:

$S_{net}=\frac{1}{N}\sum_{i\in G} S_{i}$,

for weighted network, $S_{i}$ is the sum of the edge weights $w_{ij}$ linking to node $i$: $S_{i}^{W}=\sum_{j\in G} w_{ij}$, and for binarized network, $S_{i}^{B}=\sum_{j\in G} a_{ij}$ ($a_{ij}=1$, if non-zero edge). The connectivity strength of a network is the average of the nodal connectivity strength of all the nodes in the network.

Small-world properties

Small-world networks, characterized by highly local clustering and short path length linking network nodes, were originally proposed by Watts and Strogatz [[16](#_ENREF_16)]. The clustering coefficient $C_{net}$ is the average of the clustering coefficient across all nodes:

$C_{net}=\frac{1}{N}\sum_{i\in G} C_{i}$,

where $C_{i}$ expresses the likelihood that the neighbors of node $i$ are connected. For weighted network, $C_{i}$ is defined as: $C_{i}^{W}=\frac{\sum_{j,h\in G} \left(w_{ij}w_{ih}w_{jh}\right)^{1/3}}{k_{i}\left(k_{i}-1\right)}$, and for binarized network, $C_{i}^{B}=\frac{2E_{i}}{k_{i}\left(k_{i}-1\right)}$ ,$k_{i}$ is the degree of node $i$, $E_{i}$ is the number of edges in the subgraph constructed by the neighbors of node $i$. The clustering coefficient $C_{net}$ quantifies the extent of the local interconnectivity or cliquishness of information transfer on the network [[16](#_ENREF_16)].

The path length between nodes $i$ and $j$ is defined as the sum of the edge lengths along the path. For weighted network, each edge’s length was obtained by computing the reciprocal of the edge weight, $1/w_{ij}$. The shortest path length $L_{ij}$ between nodes $i$ and $j$ is the length of the path with the shortest length between the two nodes. The characteristic path length $L_{net}$ was measured using a ‘harmonic mean’ length between pairs [[17](#_ENREF_17)], to overcome the problem of possibly disconnected network components. Formally, $L_{net}$ is the reciprocal of the average of the reciprocals:

$L_{net}=\frac{1}{1/\left(N\left(N-1\right)\right)\sum_{i=1}^{N} \sum_{j\neq i}^{N} 1/L_{ij}}$.

The characteristic path length $L_{net}$ quantifies the extent of global efficiency or capability for parallel information propagation of the network.

For each individual network, the normalized clustering coefficient $\gamma=C_{net}/C_{random}$ and the normalized characteristic path length $\lambda=L_{net}/L_{random}$ were calculated respectively, where $C_{random}$ and $L_{random}$ are the mean clustering coefficient and the mean characteristic path length of 100 comparable random networks keeping the same number of nodes, edges and degree distribution as this individual network [[18](#_ENREF_18)]. For weighted network, the corresponding weights are redistributed. A small-world network has similar path length but higher clustering than a random network, that is $\gamma>1$, $\lambda\approx1$ [[16](#_ENREF_16)]. The two conditions can also be summarized into a simple quantitative measurement, the small-worldness, $\sigma=\gamma/\lambda>1$ [[19](#_ENREF_19),[20](#_ENREF_20)].

**Regional properties**

The nodal connectivity strength $S_{i}$ quantifies the total level of connectivity of a node [[15](#_ENREF_15)]. The nodal efficiency $E_{i}$ is defined as the inverse of the mean harmonic shortest path length between this node and the others in the network [[21](#_ENREF_21)]:

$E_{i}=\frac{1}{N-1}\sum_{i\neq j\in G} \frac{1}{L_{ij}}$,

which quantifies the importance of the nodes for the communication within the network [[22](#_ENREF_22)].

The betweenness centrality $B_{i}$ is the fraction of all shortest paths in the network that contain this node [[23](#_ENREF_23)]. Here, we calculated the normalized betweenness centrality as: $b_{i}=B_{i}/\left[\left(N-1\right)\left(N-2\right)\right]$, which captures the influence of a node over information flow between other nodes in the network.

**Coupling between functional and structural connectivity networks**

For each subject, we investigated the coupling between functional and structural connectivity networks. The coupling analysis was constrained by the edges with non-zero structural connectivity, similar to our previous study [[2](#_ENREF_2)]. In detail, we extracted the non-zero structural connectivities to produce a vector of structural connectivity values. Then, the values were resampled into a Gaussian distribution [[12](#_ENREF_12),[13](#_ENREF_13)]. The corresponding functional connectivities were also extracted to form a vector of functional connectivity values. The Pearson’s correlation between these two vectors was computed to quantify the coupling between functional and structural connectivity networks.

**Statistical analysis**

We first computed global topological properties ($S_{net}$, $\gamma$, $\lambda$, $\sigma$) of functional and structural connectivity networks for each subject on a range of cost thresholds (0.18≤cost≤0.39). Here, the cost was computed as the ratio of the number of actual connections divided by the maximum possible number of connections in the network. The lower cost threshold was selected to ensure all brain networks were fully connected, and the upper threshold was the maximum cost attained by structural brain networks. We also calculated the area under the curve (AUC) for global topological properties, which provides a summarized scalar for brain topological properties independent of single threshold selection. The AUC analysis is sensitive to detection of topological alterations of brain diseases and has been adopted in previous studies [[21](#_ENREF_21),[24](#_ENREF_24)]. Then, a nonparametric permutation test method was performed [[14](#_ENREF_14),[25](#_ENREF_25)] to detect the significant group differences in global topological properties. Accordingly, we computed the between-group differences of topological properties. To test the null hypothesis that the observed group differences could occur by chance, we then randomly reallocated all global topological properties into two groups and recomputed the differences between the two randomized groups. This procedure was repeated 5000 times. We assigned a *p*-value to the between-group difference by computing the proportion of differences exceeding the null distribution values. A significance threshold of *p*<0.05 (FDR-corrected) was used for testing global topological properties, except AUC of each global topological property for which an uncorrected threshold of *p*<0.01 was used since no multiple comparisons were performed. Of note, before the permutation tests, age and gender for each network properties were regressed out as covariates by multiple linear regression analyses.

To detect differences of regional properties, the same permutation framework was performed on the AUC of each regional property ($S_{i}$, $E_{i}$ and $b_{i}$). A significance threshold of *p*=0.01 (uncorrected) was used. To address the problem of multiple comparisons, we set the significance threshold level at *p*<0.01, Bonferroni-corrected for the number of brain regions.

Furthermore, coupling of functional and structural connectivity networks was compared by using permutation tests. Testing was performed on the difference of functional-structural connectivity coupling strength.

**References:**

1. Tzourio-Mazoyer N, Landeau B, Papathanassiou D, Crivello F, Etard O, et al. (2002) Automated anatomical labeling of activations in SPM using a macroscopic anatomical parcellation of the MNI MRI single-subject brain. Neuroimage 15: 273-289.

2. Zhang Z, Liao W, Chen H, Mantini D, Ding JR, et al. (2011) Altered functional-structural coupling of large-scale brain networks in idiopathic generalized epilepsy. Brain 134: 2912-2928.

3. Ding JR, Chen H, Qiu C, Liao W, Warwick JM, et al. (2011) Disrupted functional connectivity in social anxiety disorder: a resting-state fMRI study. Magn Reson Imaging 29: 701-711.

4. Power JD, Barnes KA, Snyder AZ, Schlaggar BL, Petersen SE (2012) Spurious but systematic correlations in functional connectivity MRI networks arise from subject motion. Neuroimage 59: 2142-2154.

5. Van Dijk KR, Sabuncu MR, Buckner RL (2012) The influence of head motion on intrinsic functional connectivity MRI. Neuroimage 59: 431-438.

6. Salvador R, Suckling J, Coleman MR, Pickard JD, Menon D, et al. (2005) Neurophysiological architecture of functional magnetic resonance images of human brain. Cereb Cortex 15: 1332-1342.

7. Wang R, Beener T, Sorensen AG, Weeden VJ (2007) Diffusion toolkit: a software package for diffusion imaging data processing and tractography. Proc Intl Soc Mag Reson Med 15: 3720.

8. Liao W, Zhang Z, Pan Z, Mantini D, Ding J, et al. (2010) Default mode network abnormalities in mesial temporal lobe epilepsy: A Study combining fMRI and DTI. Hum Brain Mapp: DOI: 10.1002/hbm.21076.

9. Gong G, He Y, Concha L, Lebel C, Gross DW, et al. (2009) Mapping anatomical connectivity patterns of human cerebral cortex using in vivo diffusion tensor imaging tractography. Cereb Cortex 19: 524-536.

10. Li Y, Liu Y, Li J, Qin W, Li K, et al. (2009) Brain anatomical network and intelligence. PLoS Comput Biol 5: e1000395.

11. Hagmann P, Cammoun L, Gigandet X, Meuli R, Honey CJ, et al. (2008) Mapping the structural core of human cerebral cortex. PLoS Biol 6: e159.

12. Hagmann P, Sporns O, Madan N, Cammoun L, Pienaar R, et al. (2010) White matter maturation reshapes structural connectivity in the late developing human brain. Proc Natl Acad Sci U S A 107: 19067-19072.

13. Honey CJ, Sporns O, Cammoun L, Gigandet X, Thiran JP, et al. (2009) Predicting human resting-state functional connectivity from structural connectivity. Proc Natl Acad Sci U S A 106: 2035-2040.

14. van den Heuvel MP, Mandl RC, Stam CJ, Kahn RS, Hulshoff Pol HE (2010) Aberrant frontal and temporal complex network structure in schizophrenia: a graph theoretical analysis. J Neurosci 30: 15915-15926.

15. Rubinov M, Sporns O (2010) Complex network measures of brain connectivity: Uses and interpretations. Neuroimage 52: 1059-1069.

16. Watts DJ, Strogatz SH (1998) Collective dynamics of 'small-world' networks. Nature 393: 440-442.

17. Newman MEJ (2003) The structure and function of complex networks. Siam Review 45: 167-256.

18. Maslov S, Sneppen K (2002) Specificity and stability in topology of protein networks. Science 296: 910-913.

19. Humphries MD, Gurney K, Prescott TJ (2006) The brainstem reticular formation is a small-world, not scale-free, network. Proc Biol Sci 273: 503-511.

20. Achard S, Salvador R, Whitcher B, Suckling J, Bullmore E (2006) A resilient, low-frequency, small-world human brain functional network with highly connected association cortical hubs. J Neurosci 26: 63-72.

21. Achard S, Bullmore E (2007) Efficiency and cost of economical brain functional networks. PLoS Comput Biol 3: e17.

22. Bassett DS, Bullmore E (2006) Small-world brain networks. Neuroscientist 12: 512-523.

23. Freeman LC (1977) A set of measures of centrality based on betweenness. Sociometry 40: 35-41.

24. Broyd SJ, Demanuele C, Debener S, Helps SK, James CJ, et al. (2009) Default-mode brain dysfunction in mental disorders: a systematic review. Neurosci Biobehav Rev 33: 279-296.

25. Bassett DS, Bullmore E, Verchinski BA, Mattay VS, Weinberger DR, et al. (2008) Hierarchical organization of human cortical networks in health and schizophrenia. J Neurosci 28: 9239-9248.
